# Pruinosanones A-C, anti-inflammatory isoflavone derivatives from *Caragana pruinosa*

**DOI:** 10.1038/srep31743

**Published:** 2016-08-22

**Authors:** Chengjian Zheng, Liang Wang, Ting Han, Hailiang Xin, Yiping Jiang, Lan Pan, Xiaoguang Jia, Luping Qin

**Affiliations:** 1 Department of Pharmacognosy, School of Pharmacy, Second Military Medical University, Shanghai 200433, China; 2 Xinjiang Institute of Chinese Materia Medica and Ethnodrug, Urumqi 830002, China

## Abstract

Pruinosanone A (**1**), a novel spirochromone, was isolated from the roots of *Caragana pruinosa*. Two biogenetically related isoflavone intermediates, pruinosanones B and C (**2** and **3**), were also isolated, together with five known analogs identified as 3-hydroxy-9-methoxypterocarpan (**4**), 7,2′-dihydroxy-4′-methoxyisoflavanol (**5**), retusin-8-methylether (**6**), 7,2′-dihydroxy-8,4′-dimethoxy isoflavone (**7**) and 7,3′-dihydroxy-8,4′-dimethoxy isoflavone (**8**). The structures of **1**–**3** were elucidated based on extensive spectroscopic methods. Notably, **1** is the first example of a spirochromone possessing an unprecedented pentacyclic skeleton containing a spiro[benzo[*d*][1,3]dioxole-2,3′-chroman]-4′-one motif, which was confirmed by X-ray diffraction analysis. A plausible biosynthetic pathway for **1** was also proposed. Compounds **1**–**8** were tested for their ability to inhibit nitric oxide (NO) production in LPS-induced RAW 264.7 macrophages, and compounds **1**–**3** were the most potent inhibitors of NO production, with IC_50_ values of 1.96, 1.93 and 1.58 μM, respectively. A structure-activity relationship analysis revealed that the fused 2-isopropenyl-2,3-dihydrofuran moiety plays a vital role in the potency of these compounds. Moreover, **1** was found to significantly inhibit inducible nitric oxide synthase (iNOS) protein expression, which accounts for the potent inhibition of NO production by this spirochromone.

*Caragana pruinosa* (Leguminosae) is a dwarf shrub that is found primarily in the Xinjiang Province of China and Central Asia. Its roots are widely used for the treatment of inflammatory disorders in the folk medicine of northwestern China^1^. However, its chemical components and pharmacological effects have not been reported. Phytochemical investigations of other *Caragana* plants have confirmed the presence of stilbenoids^2^,^3^, terpenoids^4^,^5^ and flavonoids^6^,^7^, which are responsible for the medicinal use of the plants to treat inflammation, wounds, infections, hypertension, arthritis and cancer^8^.

As part of our continuing efforts focused on *Caragana* species^4^,^9^,^10^,^11^, the present study was performed to investigate the bioactive components in *C. pruinosa*, which led to the characterization of a novel spirochromone called pruinosanone A (**1**). This compound possesses a novel spiro[benzo[*d*][1,3]dioxole-2,3′-chroman]-4′-one ring system within its pentacyclic skeleton (Fig. 1). Two biogenetically related isoflavone intermediates, pruinosanones B and C (**2** and **3**), together with five known analogs (**4**–**8**), were also isolated. To the best of our knowledge, this is the first phytochemical report on this medicinal species. Herein, we report the isolation, structural elucidation and determination of the absolute configuration of the new compounds (**1**–**3**). Furthermore, a pathway for the biosynthesis of **1** from chalcone, involving two epoxidation steps, is proposed. Finally, we investigated the inhibition of nitric oxide (NO) production by compounds **1–8** in LPS-induced RAW 264.7 cells. Compounds **1–3**, which bear a fused 2-isopropenyl-2,3-dihydrofuran moiety, exhibited much better activity, with IC_50_ values ranging from 1.58 to 1.96 μM. Compound **1** was found to be a potent NO production inhibitor, remarkably suppressing NO release with an IC_50_ value of 1.96 μM while causing no cytotoxicity. This NO suppression was probably due to down-regulation of inducible nitric oxide synthase (iNOS) expression.

## Results and Discussion

### Isolation and structure elucidation

The EtOH extract of *C. pruinosa* roots was subjected to a succession of chromatographic procedures to yield three new isoflavone derivatives, named pruinosanones A-C (**1**–**3**), along with five known analogs (**4**–**8**). The structures of the known compounds were elucidated by comparing their NMR data with those reported in the literature and were identified as 3-hydroxy-9-methoxypterocarpan (**4**)^12^, 7,2′-dihydroxy-4′-methoxyisoflavanol (**5**)^13^, retusin-8-methylether (**6**)^14^, 7,2′-dihydroxy-8,4′-dimethoxy isoflavone (**7**)^15^ and 7,3′-dihydroxy-8,4′-dimethoxy isoflavone (**8**)^16^.

**Figure 1:**
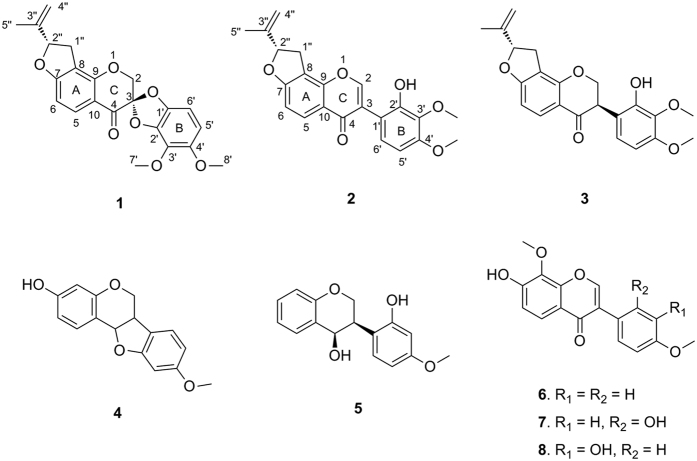
Structures of isolated compounds (**1**–**8**).

**Figure 2:**
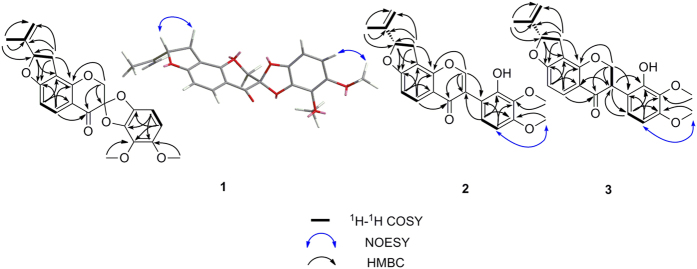
Key ^1^H-^1^H COSY, HMBC and NOESY correlations of **1**–**3**.

Pruinosanone A (**1**), obtained from *C. pruinosa* as optically active, colorless needles (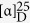 + 25°), possesses the molecular formula C_22_H_20_O_7_ (13 degrees of unsaturation), which was deduced from HRESIMS analysis ([M + H]^+^ at *m/z* 397.1269), ^13^C NMR data, and DEPT spectra (see Supplementary Information). The ^1^H NMR spectrum (Table 1) of **1** displayed signals for two pairs of ortho-aromatic protons [*δ*_H_ 7.89 (1H, d, *J* = 8.4 Hz, H-5) and *δ*_H_ 6.64 (1H, d, *J* = 8.4 Hz, H-6); *δ*_H_ 6.49 (1H, d, *J* = 8.4 Hz, H-6′) and *δ*_H_ 6.37 (1H, d, *J* = 8.4 Hz, H-5′)], an isopropenyl group [*δ*_H_ 1.80 (3H, s, H-5″), *δ*_H_ 5.12 (1H, d, *J* = 3.0 Hz, H-4″) and *δ*_H_ 4.98 (1H, d, *J* = 3.0 Hz, H-4″)], an oxygenated methine [*δ*_H_ 5.37 (1H, dd, *J* = 10.2, 7.8 Hz, H-2″)], an oxygenated methylene [*δ*_H_ 4.64 (1H, d, *J* = 12.6 Hz, H-2) and *δ*_H_ 4.63 (1H, d, *J* = 12.6 Hz, H-2)], a benzylic methylene [1H, *δ*_H_ 3.37 (dd, *J* = 16.2, 10.2 Hz, H-1″), *δ*_H_ 3.04 (1H, dd, *J* = 16.2, 7.8 Hz, H-1″)], and two methoxy groups [*δ*_H_ 4.02 (3H, s, OCH_3_-3′) and *δ*_H_ 3.82 (3H, s, OCH_3_-4′)]. The ^13^C and DEPT spectra revealed the presence of 22 carbon resonances, which were divided into 3 methyls, 3 methylenes, 5 methines, and 11 quaternary carbons. The ^1^H–^1^H COSY spectrum (Fig. 2) of **1** revealed the presence of H-3″/H-4″ and H-1″/H-2″ fragments, combined with the HMBC correlations between H-1″, H-5″/C-2″ (*δ*_C_ 88.1), C-3″ (*δ*_C_ 142.8), C-4″ (*δ*_C_ 112.9), suggesting the presence of an 3-oxygenated 1-isopentenyl group. In addition to two methoxy groups, the remaining 15 carbon resonances were observed to be fairly close to those of isoflavanones, with two characteristic signals at *δ*_C_ 70.5 (C-2, oxygenated methylene) and *δ*_C_ 180.7 (C-4, carbonyl), except for the distinguished geminal coupling of the H-2 [*δ*_H_ 4.64 (1H, d, *J* = 12.6 Hz) and *δ*_H_ 4.63 (1H, d, *J* = 12.6 Hz)] neighboring the quaternary carbon C-3 (*δ*_C_ 106.0), with a large down-field shift in **1**. C-3 was assigned to be an acetal carbon deduced from two more oxygen atoms that remained according to the molecular formula, which led to the formation of a spiro system between rings C and B. The HMBC experiment also indicated correlations between H-2/C-3, C-4, H-5′/C-1′, and H-6′/C-2′. Furthermore, the HMBC correlations (Fig. 2) between H-2″/C-7 and C-8 revealed that the oxygenated 1-isopentenyl group was attached at C-8. The downfield shifts of C-2″ and C-7 indicated that C-2″ and C-7 are linked through an oxygen bridge, thus forming a furan ring. In addition, the HMBC spectrum of compound **1** displayed long-range correlations of H-8′, H-6′/C-4′ and H-7′, H-5′/C-3′, combined with a NOESY cross peak between H-8′ and H-5′, indicating that one methoxy group was linked to C-3′ and the other to C-4′. Based on these data, we hypothesized that compound **1** likely possesses an unprecedented pentacyclic skeleton containing a spiro[benzo[*d*][1,3]dioxole-2,3′-chroman]-4′-one motif. The planar structure that was established is shown in Fig. 1, and this compound was named pruinosanone A.

The relative stereochemistry of **1** was deduced on the basis of its NOESY spectroscopic data (Fig. 2). The NOESY correlations of H-2″ with H-1″β indicated that these two protons are located on the same face. The configuration of **1** at C-2″ was thereby established, whereas no obvious NOESY correlation was detected for determining the relative configuration of another chiral carbon at C-3.

**Figure 3:**
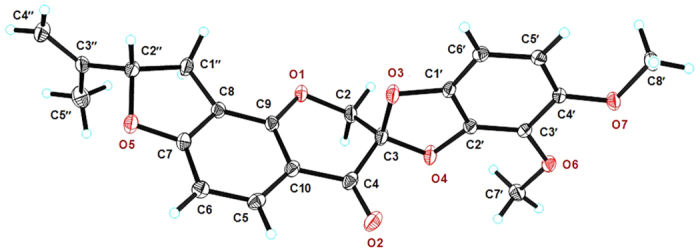
Single-crystal X-ray diffraction and ORTEP drawing of pruinosanone A (**1**).

Fortunately, **1** was crystallized by slow evaporation from a solution in a mixture of CH_3_OH/CH_2_Cl_2_ (1:1) over 7 days. The resulting crystals were suitable in size and quality for single-crystal X-ray analysis using anomalous dispersion with Cu Kα radiation, which revealed an absolute structure parameter (Flack’s x) of 0.03(7)^17^,^18^, allowing unambiguous assignment of the complete absolute configuration of **1** as a 3*S* and 2″*S* configuration (Fig. 3). The structure of pruinosanone A (**1**) was therefore defined as (2*S*, 8′*S*)-4,5-dimethoxy-8′-(prop-1-en-2-yl)-8′,9′-dihydrospiro[benzo[*d*][1,3]dioxole-2,3′-furo[2,3-*h*]chromen]-4′(2′*H*)-one.

Compound **2**, named pruinosanone B, was obtained as a yellow, amorphous powder. The positive HRESIMS data ([M + H]^+^ at *m/z* 381.1343, calcd 381.1338) indicated the molecular formula of **2** to be C_22_H_20_O_6_. Its ^1^H and ^13^C NMR spectra (Table 2) were characterized by data typical for an isoflavone skeleton and were analogous to those of **1**, except for the major differences in the signals corresponding to C-2 and H-2 (*δ*_H_ 8.24, s, 1H and *δ*_C_ 154.6 in **2**; *δ*_H_ 4.63, 4.64, d, *J* = 12.6, 2H and *δ*_C_ 70.5 in **1**) and the appearance of several slightly shifted signals from the B-ring. These data indicated that **2** was an isoflavone derivative of **1**, which was confirmed by detailed HMBC analysis, particularly the HMBC correlations from H-2 (*δ*_H_ 8.24, s, 1H) to C-4 (*δ*_C_ 175.3), C-1′ (*δ*_C_ 113.9) and from H-6′ (*δ*_H_ 6.90, d, *J* = 8.4, 1H) to C-3 (*δ*_C_ 122.2) (Fig. 2). The signals for two pairs of ortho-aromatic protons in **2** were nearly the same as those in **1**, revealing an identical substitution pattern in rings A and B for **2** and **1**. The NOESY correlations were also similar to those of **1**. The absolute configuration at C-2″ could be assigned by analogy to the configuration of **1** from a biosynthetic view and was confirmed by the similar signals for H-2″ [*δ*_H_ 5.54 (dd, 9.6, 7.8) in **2**; *δ*_H_ 5.37 (dd, 10.2, 7.8) in **1**] and C-2″ (*δ*_C_ 87.8 in **2**; *δ*_C_ 88.1 in **1**). The structure of pruinosanone B (**2**) was therefore defined as (2″*S*)-8-(2-methylbut-1-en-4-yl)-7,2″-epoxy-2′-hydroxy-3′,4′-dimethoxyisoflavone.

Compound **3**, named pruinosanone C, was obtained as a yellow powder. The molecular formula of **3** was determined to be C_22_H_22_O_6_ by positive HRESIMS ([M + H]^+^ at *m/z* 383.1492, calcd 383.1494). Its ^1^H NMR and ^13^C NMR data are summarized in Table 2 and suggest that the compound has most of the same structural features as **2**, except that compound **3** has one less degree of unsaturation than **2**. Considering the molecular weights of **3** and **2**, compound **3** was considered to be a 2,3-hydrogenation derivative of **2**, which was further confirmed by detailed HMBC, COSY and NOESY examination. The equatorial orientation of the B-ring was verified by the NMR coupling constant between the *trans*-diaxial H-2β and H-3 of *ca*. 11 Hz^19^. Furthermore, circular dichroism (CD) was used to establish the absolute configuration of C-3 as 3*R* based on the positive cotton effect (CE) of the n → π^*^ carbonyl absorption band at 329 nm^20^. Thus, pruinosanone C (**3**) was concluded to be (3*R*, 2″*S*)-8-(2-methylbut-1-en-4-yl)-7,2″-epoxy-2′-hydroxy-3′,4′-dimethoxyisoflavanone.

**Figure 4:**
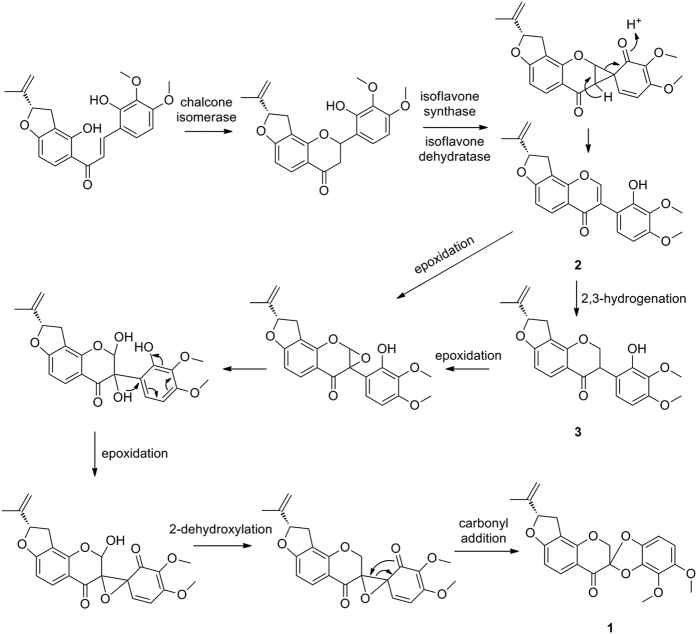
Proposed biosynthetic pathway for pruinosanone A (**1**).

The biosynthetic pathways for **1**–**3** were proposed to start from a chalcone derivative (Fig. 4). This pathway involves two key epoxidation steps^21^ that occur between C-2/C-3 and C-3/C-1′, respectively, followed by an intermolecular carbonyl addition reaction to form a five-membered spiro-heterocycle between rings C and B.

### Ability of the compounds to inhibit LPS-induced NO production

**Figure 5:**
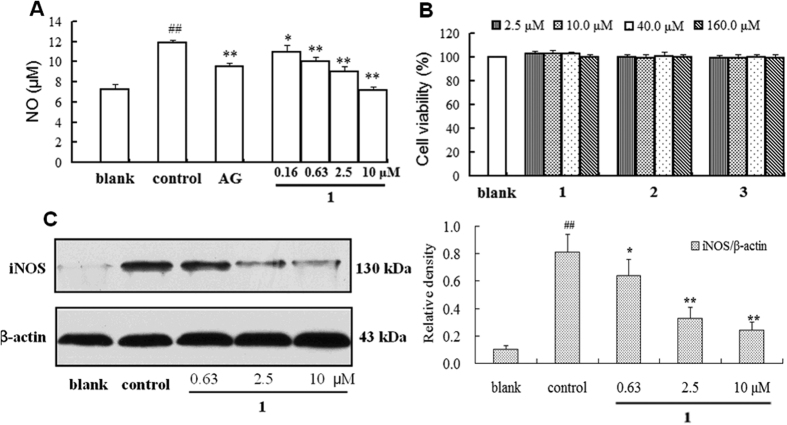
(**A**) Inhibitory effect of pruinosanone A (**1**) on NO production in LPS-induced RAW 264.7 macrophages (Control: 1 μg/mL LPS; AG: aminoguanidine, 20 μΜ, as a positive control). (**B**) Effects of pruinosanones A-C (**1–3**) on the cell viability of RAW 264.7 cells. (**C**) Western blot analysis of iNOS protein. β-actin was used as a loading control. ^##^*p* < 0.01 vs blank; *p < 0.05 vs control; **p < 0.01 vs control.

NO acts as a host defense mechanism by damaging pathogenic DNA and is also a regulatory molecule with homeostatic activities^22^. However, an excess production of NO in biological systems gives rise to various diseases, such as inflammation, cancer, and atherosclerosis^23^. Therefore, substances that inhibit NO release may be of therapeutic benefit in various disorders induced by pathological levels of NO^21^. Compounds **1**–**8** were tested for their ability to inhibit NO production in LPS-induced RAW 264.7 macrophages according to a previously described method^24^,^25^. The results (Table 3) indicated that compounds **1–3**, which have a 2-isopropenyl-2,3-dihydrofuran moiety fused with ring A, possessed much better inhibitory activity, with IC_50_ values ranging from 1.58 to 1.96 μM, than compounds **4**–**8**, which had IC_50_ values >100 μM. This suggests that the presence of a fused 2-isopropenyl-2,3-dihydrofuran ring plays a vital role in the potency of these compounds. Pruinosanone A (**1**) was a potent inhibitor of NO production, remarkably suppressing NO release in a dose-dependent manner (Fig. 5A), with an IC_50_ value of 1.96 μM, which is much less than that of the positive control aminoguanidine (AG, IC_50_ 20.13 μM).

### Cell viability

Cell viability was determined using the CCK-8 method to evaluate whether the inhibition of NO production was due to the cytotoxicity of the tested compounds. It was found that none of the concentrations used in this experiment were cytotoxic (Fig. 5B). Thus, the inhibitory activity of these isoflavone derivatives was not due to their cytotoxic properties but to their ability to suppress NO production, which merited further study regarding the precise site and mechanism of action of these compounds.

### Western blot analysis

Nitric oxide synthases (NOSs) play a very important role in catalyzing the production of NO from L-arginine. Inflammatory mediators, such as IL-1, TNF-α, and LPS, stimulate the expression of the inducible isoform of NOS (iNOS) in rodent macrophages, which leads to the prolonged production of large amounts of NO, a characteristic of many inflammatory diseases^26^,^27^,^28^,^29^. To elucidate the underlying mechanism of these compounds in the inhibition of NO, pruinosanone A (**1**) was selected to investigate the effect of the compound on iNOS protein expression in LPS-induced RAW 264.7 cells. According to the results, pruinosanone A was found to significantly down-regulate the expression of iNOS in a dose-dependent manner, which accounts for the potent inhibitory activity of compound **1** against NO production (Fig. 5C).

In conclusion, pruinosanone A (**1**) is the first pentacyclic spirochromone containing a spiro[benzo[*d*][1,3]dioxole-2,3′-chroman]-4′-one motif, which has never been observed before for any chromone. Therefore, this structure represents a new carbon skeleton. The structure of **1** provides not only an interesting synthetic target but also a potent inhibitor of NO release. Further studies on this molecule will provide valuable insights into the development of anti-inflammatory drugs.

## Methods

### General experimental procedures

Optical rotations were acquired with a Perkin-Elmer 341 polarimeter. The UV spectra were acquired using a Varian Cary Eclipse 300 spectrophotometer, while the IR spectra were recorded on a Bruker Vector 22 spectrometer with KBr pellets. The NMR spectra were recorded on a Bruker Avance 600 NMR spectrometer with TMS as an internal standard. The HRESIMS measurements were obtained with a Q-TOF Micromass spectrometer (Waters, USA). X-ray crystallographic analysis was performed with a Bruker SMART APEX (II)-CCD diffractometer with Cu Kα radiation (λ = 1.54178 Å). The materials for the CC were silica gel (100–200 mesh; Huiyou Silical Gel Development Co. Ltd., Yantai, China), silica gel H (10–40 μm; Yantai), Sephadex LH-20 (40–70 μm; Amersham Pharmacia Biotech AB, Uppsala, Sweden), and YMC-GEL ODS-A (50 μm; YMC, Milford, MA). Semi-preparative HPLC was conducted on an Agilent 1200 instrument using an Eclipse XDB-C_18_ column (5 μm, 9.4 × 250 mm). Preparative TLC (0.4–0.5 mm) was conducted on glass plates precoated with silica gel GF254 (Yantai).

### Plant material

*C. pruinosa* roots were collected from Urumuchi, Xinjiang, P. R. China and authenticated by Prof. Xiao-Guang Jia, Xinjiang Institute of Chinese Materia Medica and Ethnodrug (Urumuchi, China). A voucher specimen of this plant was kept at the Herbarium of the Department of Pharmacognosy, School of Pharmacy, Second Military Medical University, Shanghai, P.R. China (No. *201203).

### Extraction and isolation

The air-dried and pieced roots of *C. pruinosa* (11.5 kg) were extracted with 80% EtOH (×3), with each extraction period lasting 2 h. The solvent was removed under reduced pressure, and the residue (1.2 kg) was suspended in H_2_O and partitioned sequentially with petroleum ether, EtOAc, and *n*-butanol to afford four fractions, a petroleum ether fraction (PEF, 33.9 g), an EtOAc fraction (EF, 226.9 g), an *n*-butanol fraction (BF, 100.9 g) and a remaining water fraction (WF). In our preliminary study, the EtOAc-soluble fraction exhibited notable anti-inflammatory activity *in vitro* and therefore was selected for investigation in the present study.

The EtOAc fraction (EF, 226.9 g) was subjected to CC on silica gel (200–300 mesh, 900 g) and eluted successively with a gradient of petroleum ether-EtOAc mixtures (50:1, 20:1, 10:1, 5:1, 3:1, 1:1, and 0:1, v/v) to afford fractions A-G. Fraction C (5.0 g) was further fractionated by column chromatography on silica gel (200–300 mesh) employing a petroleum ether-EtOAc mixture (20:1) as the eluent to provide five fractions (C.1-C.5). Fraction C.3 (400 mg) was rechromatographed on Sephadex LH-20 resin with MeOH-H_2_O (80:20, v/v) followed by preparative TLC to yield compounds **2** (7 mg) and **3** (6 mg). Fraction C.4 (500 mg) was further separated on Sephadex LH-20 resin with MeOH-H_2_O (80:20, v/v) followed by semi-preparative HPLC with MeOH-H_2_O (75:25, v/v) as the eluent to yield compound **1** (8 mg), which was further crystallized by slow evaporation from a solution of a CH_3_OH/CH_2_Cl_2_ (1:1) mixture over 7 days.

**Pruinosanone A (1):** colorless needles; m.p. 123–124 °C; 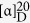 +25° (*c* 0.08, methanol); IR (KBr) *v*_*max*_ 2924, 2852, 1610, 1511, 1464, 1384, 1084; ^1^H-NMR (CDCl_3_, 600 MHz) and ^13^C-NMR (CDCl_3_, 150 MHz) spectra (Table 1); HRESIMS *m*/*z* 397.1269 [M + H]^+^ (calcd for C_22_H_21_O_7_, 397.1287).

**Pruinosanone B (2):** yellow amorphous powder; 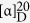 +42.7° (*c* 0.06, methanol); IR (KBr) *v*_*max*_ 3394, 2925, 2854, 1610, 1509, 1458, 1243, 1105, 1036, 829; ^1^H-NMR (DMSO-*d*_6_, 600 MHz) and ^13^C-NMR (DMSO-*d*_6_, 150 MHz) spectra (Table 2); HRESIMS *m*/*z* 381.1341 [M + H]^+^ (calcd for C_22_H_21_O_6_, 381.1338).

**Pruinosanone C (3):** yellow amorphous powder; 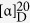 +54.6° (*c* 0.04, methanol); CD (*c* 0.04, methanol) [*Δε*]_329_ = +9.6; IR (KBr) *v*_*max*_ 3421, 2924, 2853, 1654, 1608, 1508, 1466, 1258, 1094; ^1^H-NMR (CD_3_OD, 600 MHz) and ^13^C-NMR (CD_3_OD, 150 MHz) spectra (Table 2); HRESIMS *m*/*z* 383.1492 [M + H]^+^ (calcd for C_22_H_23_O_6_, 383.1494).

### X-ray crystallographic analysis of pruinosanone A (1)

Upon crystallization from CH_3_OH/CH_2_Cl_2_ (1:1) using the vapor diffusion method, colorless crystals were obtained for **1**. A crystal was separated from the sample and mounted on a glass fiber. X-ray crystallographic analysis was carried out on a Bruker SMART APEX (II)-CCD diffractometer with Cu Kα radiation (λ = 1.54178 Å). Structure solution and refinement were performed with the SHELXL-97 program. The crystal data for pruinosanone A (**1**) are as follows: Empirical formula: C_22_H_20_O_7_; Formula weight: 396.38; Crystal system: monoclinic; Space group: *P*2_1_; Crystal size: 0.250 mm × 0.200 mm × 0.100 mm; Unit cell dimensions: *a* = 9.58860(10) Å, *b* = 9.43910(10) Å, *c* = 10.7943(2) Å, *α* = 90°, *β* = 109.7170(10)°, *γ* = 90°, *V* = 919.69(2) Å^3^; Index ranges: −11 ≤ *h* ≤ 11, −11 ≤ *k* ≤ 9, −13 ≤ *l* ≤ 13; *θ* range for the data collection was from 4.351° to 69.814°; *Z* = 2; *Dc* = 1.431 g/cm^3^; *F* (000) = 416; Refinement method: Full-matrix least-squares on *F*^*2*^; Goodness-of-fit on *F*^*2*^: 1.183; Final R indices [*I* > *2σ* (*I*)]: *R*_*1*_ = 0.0324, *wR*_*2*_ = 0.0834; *R* indices (all data): *R*_*1*_ = 0.0338, *wR*_*2*_ = 0.0927; Largest differences in peak and hole: 0.377 and −0.247 e/Å^−3^; absolute structure parameter (Flack’s x) of 0.03(7).

The crystallographic data for the structure of pruinosanone A (**1**) reported in this paper have been deposited at the Cambridge Crystallographic Data Center as supplementary publication number CCDC 1058372. Copies of the data can be obtained free of charge via www.ccdc.cam.ac.uk/data_request/cif (or from the Cambridge Crystallographic Data Centre, 12 Union Road, Cambridge CB21EZ, UK; fax: (144) 1223-336-033; e-mail: deposit@ccdc.cam.ac.uk).

### Inhibition of LPS-induced NO production

RAW 264.7 macrophages were seeded in 96-well plates (10^5^ cells/well). The cells were co-incubated with the isolated compounds and LPS (1 μg/mL) for 24 h. The amount of NO was assessed by determining the nitrite concentration in the cultured RAW 264.7 macrophage supernatants with the Griess reagent. Aliquots of the supernatants (100 μL) were incubated in sequence with 50 μL of 1% sulfanilamide and 50 μL of 0.1% naphthylethylenediamine in a 2.5% phosphoric acid solution. The absorbance at 548 nm was read using a microplate reader (POLARstar).

### Cell viability

Cell viability was assessed using the mitochondrial respiration-dependent MTT reduction method. After transferring the required supernatant to another plate for the Griess assay, the remaining supernatant was aspirated from the 96-well plates, and 100 μL of fresh medium containing 2 mg/mL of MTT was added to each well. The cells were incubated at 37 °C in a humidified atmosphere containing 5% CO_2_. After incubating for 3 h, the medium was removed, and the violet crystals of formazan in the viable cells were dissolved in DMSO. The absorbance at 570 nm was measured using a microplate reader.

### Western blot analysis

The murine RAW 264.7 cell line was seeded at an initial density of 2 × 10^6^ cells/well in 6-well tissue culture plates and incubated overnight. The cells were exposed to *Escherichia coli* LPS (1 μg/mL; Sigma) for 24 h in the presence or absence of the tested compounds. These compounds were dissolved in DMSO at an initial concentration of 10 mM and diluted to an appropriate concentration using culture medium; the final concentration of DMSO was adjusted to ≤0.01%. Beta-actin (β-actin) was used to ensure that the amounts of protein were equal in each lane. Protein samples were collected and prepared as described previously^30^, and the iNOS expression levels were investigated using western blot analysis. Briefly, samples containing equal quantities of protein (50 μg) were subjected to SDS/20%-polyacrylamide gel electrophoresis, and the separated proteins were electrophoretically transferred to nitrocellulose (NC) membranes. The resulting NC membranes were incubated with a blocking solution and probed with an antibody specific to the iNOS protein (1:1000 dilution; Cell Signaling). Then, the antibodies were visualized using an ECL detection kit (Western Lightning Chemiluminescence Reagent Plus, PerkinElmer).

## Additional Information

**How to cite this article**: Zheng, C. *et al*. Pruinosanones A-C, anti-inflammatory isoflavone derivatives from *Caragana pruinosa*. *Sci. Rep*. **6**, 31743; doi: 10.1038/srep31743 (2016).

## Supplementary Material

Supplementary Information

## Figures and Tables

**Table 1:** ^1^H and ^13^C NMR data for **1** (CDCl_3_).

No.	*δ*_H_ mult. (J in Hz)	*δ*_C_	No.	*δ*_H_ mult. (J in Hz)	*δ*_C_
2a	4.64 (d, 12.6)	70.5	3″		142.8
2b	4.63 (d, 12.6)				
3		106.0	4″β	5.12 (d, 3.0)	112.9
			4″α	4.98 (d, 3.0)	
4		180.7	5″	1.8 (s)	17.0
5	7.89 (d, 8.4)	130.8	1′		142.4
6	6.64 (d, 8.4)	106.0	2′		137.6
7		167.9	3′		134.0
8		113.4	4′		147.9
9		158.1	5′	6.37 (d, 8.4)	104.7
10		113.4	6′	6.49 (d, 8.4)	101.4
1″β	3.37 (dd, 16.2, 10.2)	31.0	3′-OCH_3_	4.02 (s)	60.5
1″α	3.04 (dd, 16.2, 7.8)				
2″	5.37 (dd,10.2, 7.8)	88.1	4′-OCH_3_	3.82 (s)	56.9

**Table 2:** ^1^H and ^13^CNMR data for **2** and **3**.

No.	2		3	
	*δ*_H_ mult. (J in Hz)	*δ*_C_	*δ*_H_ mult. (J in Hz)	*δ*_C_
2β	8.24 (s)	154.6	4.73 (dd, 16.8, 11.4)	70.6
2α			4.55 (dd, 16.8, 5.4)	
3		122.2	4.24 (dd, 11.4, 5.4)	47.7
4		175.3		192.6
5	7.95 (d, 8.4)	127.6	7.83 (d, 8.4)	129.2
6	7.04 (d, 8.4)	108.8	6.57 (d, 8.4)	103.9
7		164.7		166.9
8		113.7		113.0
9		153.5		159.3
10		118.6		115.5
1′		113.9		115.3
2′		149.4		148.6
3′		136.9		136.5
4′		153.5		152.7
5′	6.58 (d, 8.4)	103.6	6.51 (d, 8.4)	103.2
6′	6.90 (d, 8.4)	126.3	6.76 (d, 8.4)	124.5
3′-OCH_3_	3.72 (s)	60.6	3.83 (s)	59.7
4′-OCH_3_	3.82 (s)	56.2	3.86 (s)	55.0
1″β	3.63 (dd, 15.6, 9.6)	31.1	3.38 (dd, 15.6, 9.6)	30.7
1″α	3.22 (dd, 15.6, 7.8)		3.00 (dd, 15.6, 7.8)	
2″	5.54 (dd, 9.6, 7.8)	87.8	5.37 (dd, 9.6, 7.8)	87.8
3″		143.6		143.7
4″β	5.14 (s)	113.1	5.12 (s)	111.2
4″α	4.98 (s)		4.97 (s)	
5″	1.77 (s)	17.2	1.80 (s)	15.7

**Table 3:** Effects of compounds **1**–**8** on NO production in LPS-stimulated RAW 264.7 cells (*n* = 3)^a^.

Compound	NO inhibition (%) at a dose of 10.0 μM; mean ± SD	IC_50_ (μM)
**1**	99.33 ± 1.51	1.96
**2**	75.98 ± 2.64	1.93
**3**	94.38 ± 2.14	1.58
**4**	2.43 ± 0.94	>100
**5**	7.51 ± 2.33	>100
**6**	6.89 ± 3.04	>100
**7**	10.60 ± 2.31	>100
**8**	11.40 ± 1.99	>100
AG^b^	23.55 ± 1.69	20.13

^a^LPS: negative control.

^b^AG: aminoguanidine, positive control.
