# Supplementary material for: Pruinosanones A-C, anti-inflammatory isoflavone derivatives from Caragana pruinosa
Source: Sci Rep. 2016 Aug 22;6:31743. doi: 10.1038/srep31743 (PMC4992842; doi:10.1038/srep31743)
Supplement: Supplementary Information [file srep31743-s1.doc]

**Supporting Information**

Pruinosanones A-C, antiinflammatory isoflavone derivatives from Caragana pruinosa

Chengjian Zheng^1^,*, Liang Wang^1^, Ting Han^1^, Hailiang Xin^1^, Yiping Jiang^1^, Lan Pan^2^, Xiaoguang Jia^2^ & Luping Qin^1^,*

^1^Department of Pharmacognosy, School of Pharmacy, Second Military Medical University, Shanghai 200433, P. R. China

^2^Xinjiang Institute of Chinese Materia Medica and Ethnodrug, Urumqi 830002, P. R. China.

*Corresponding Author: zheng_chengjian@hotmai.com (C. -J., Zheng); qinsmmu@126.com (L. -P., Qin).

**NMR spectrum for pruinosanones A-C (1-3):**

S1. ^1^H NMR (600 MHz, CDCl_3_) spectrum of pruinosanone A (**1**)

S2. ^13^C NMR spectrum (150 MHz, CDCl_3_) of pruinosanone A (**1**)

S3. DEPT spectrum (150 MHz, CDCl_3_) of pruinosanone A (**1**)

S4. HSQC spectrum of pruinosanone A (**1**)

S5. HMBC spectrum of pruinosanone A (**1**)

S6. ^1^H-^1^H COSY spectrum of pruinosanone A (**1**)

S7. NOESY spectrum of pruinosanone A (**1**)

S8. ^1^H NMR (600 MHz, DMSO-*d*_6_) spectrum of pruinosanone B (**2**)

S9. ^13^C NMR spectrum (150 MHz, DMSO-*d*_6_) of pruinosanone B (**2**)

S10. DEPT spectrum (150 MHz, DMSO-*d*_6_) of pruinosanone B (**2**)

S11. HSQC spectrum of pruinosanone B (**2**)

S12. HMBC spectrum of pruinosanone B (**2**)

S13. ^1^H-^1^H COSY spectrum of pruinosanone B (**2**)

S14. NOESY spectrum of pruinosanone B (**2**)

S15. ^1^H NMR (600 MHz, CD_3_OD) spectrum of pruinosanone C (**3**)

S16. ^13^C NMR spectrum (150 MHz, CD_3_OD) of pruinosanone C (**3**)

S17. DEPT spectrum (150 MHz, CD_3_OD) of pruinosanone C (**3**)

S18. HSQC spectrum of pruinosanone C (**3**)

S19. HMBC spectrum of pruinosanone C (**3**)

S20. ^1^H-^1^H COSY spectrum of pruinosanone C (**3**)


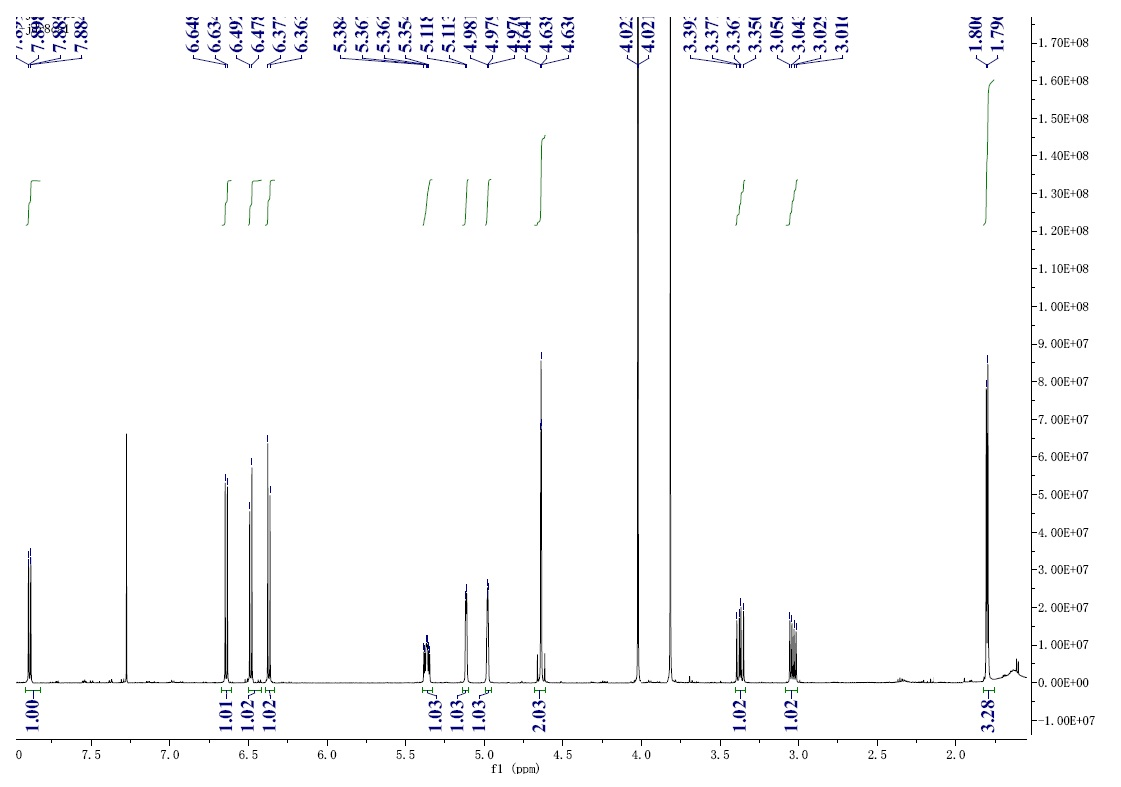


S1. ^1^H NMR (600 MHz, CDCl_3_) spectrum of pruinosanone A (**1**)


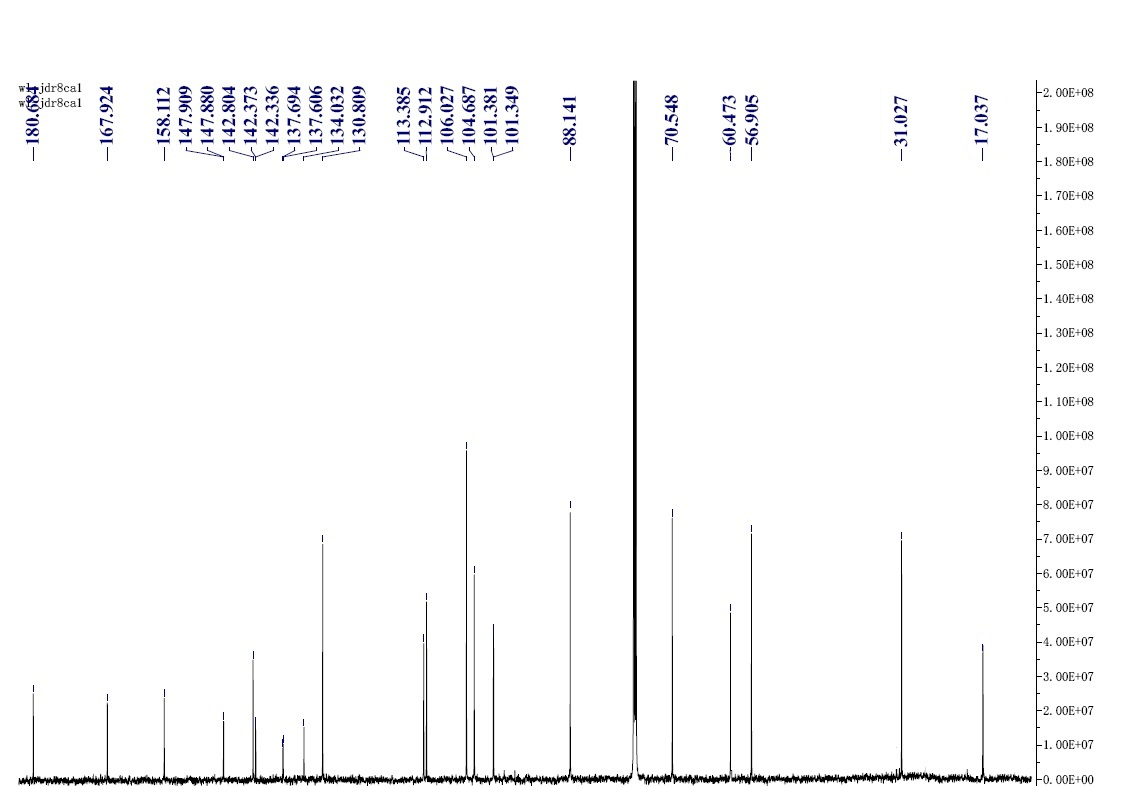


S2. ^13^C NMR spectrum (150 MHz, CDCl_3_) of pruinosanone A (**1**)


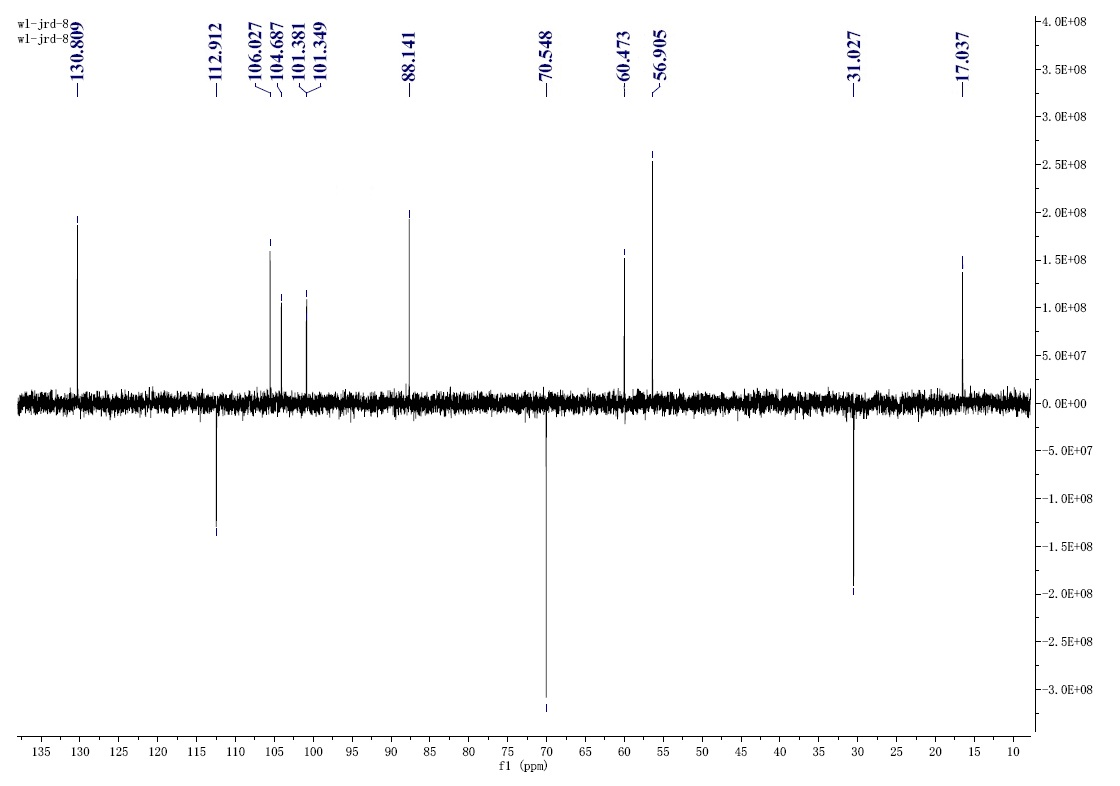


S3. DEPT spectrum (150 MHz, CDCl_3_) of pruinosanone A (**1**)


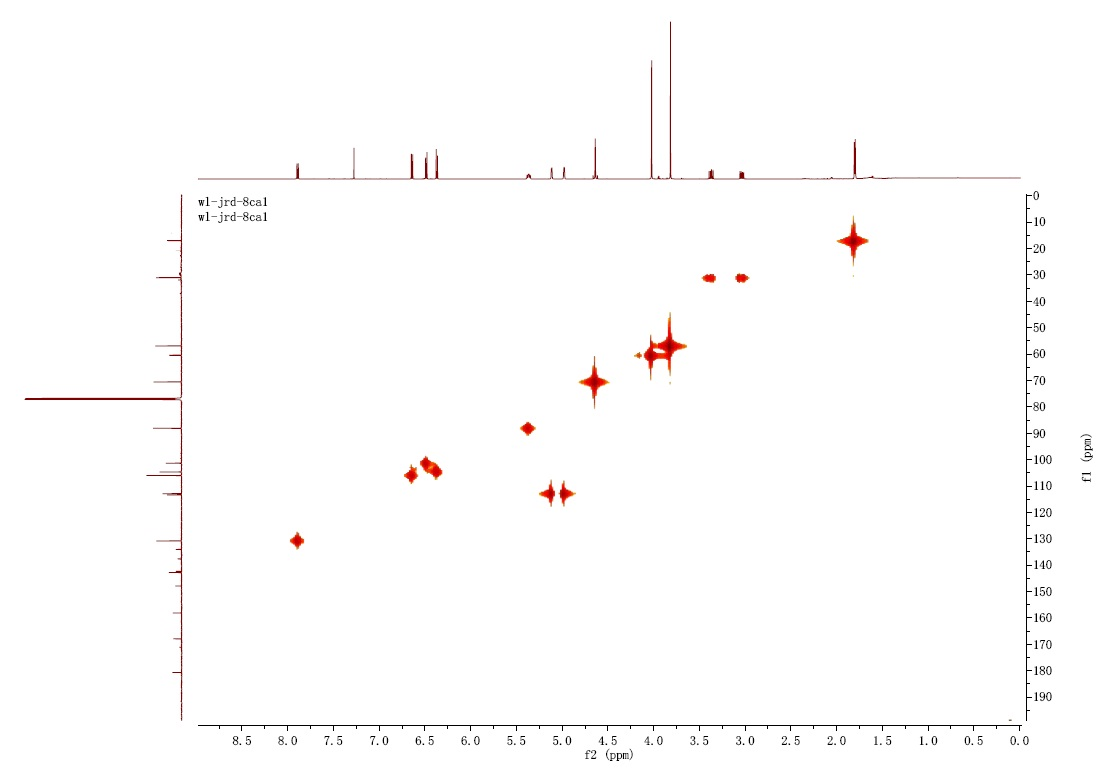


S4. HSQC spectrum of pruinosanone A (**1**)


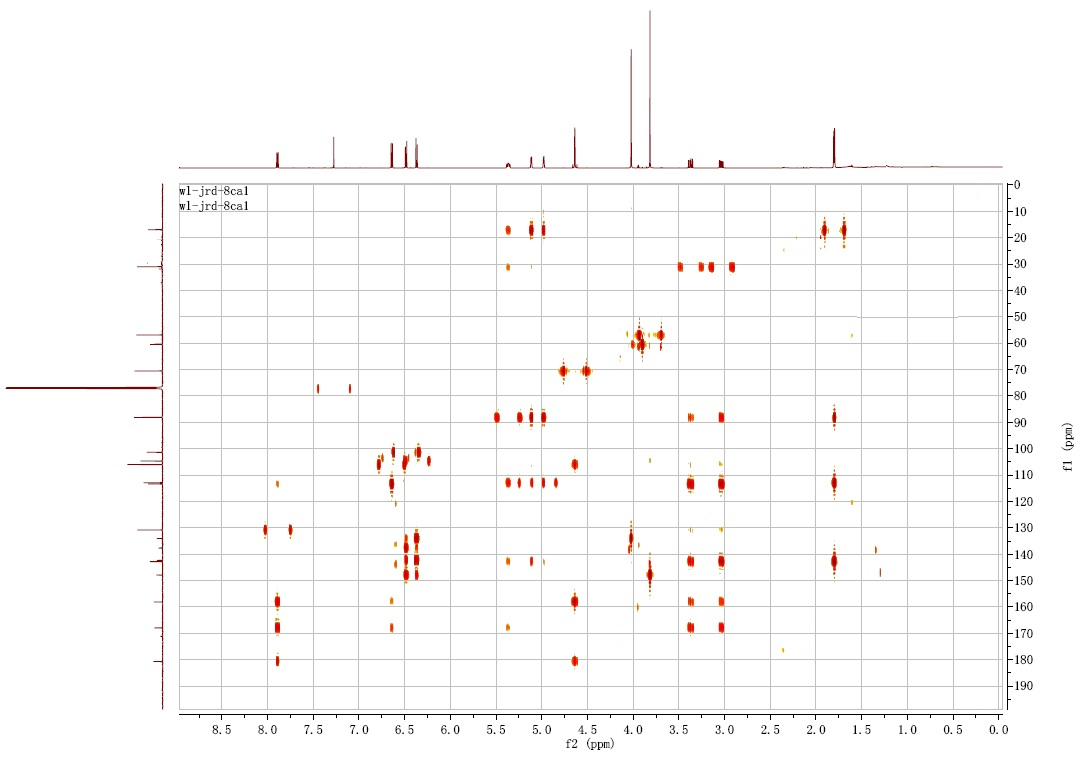


S5. HMBC spectrum of pruinosanone A (**1**)


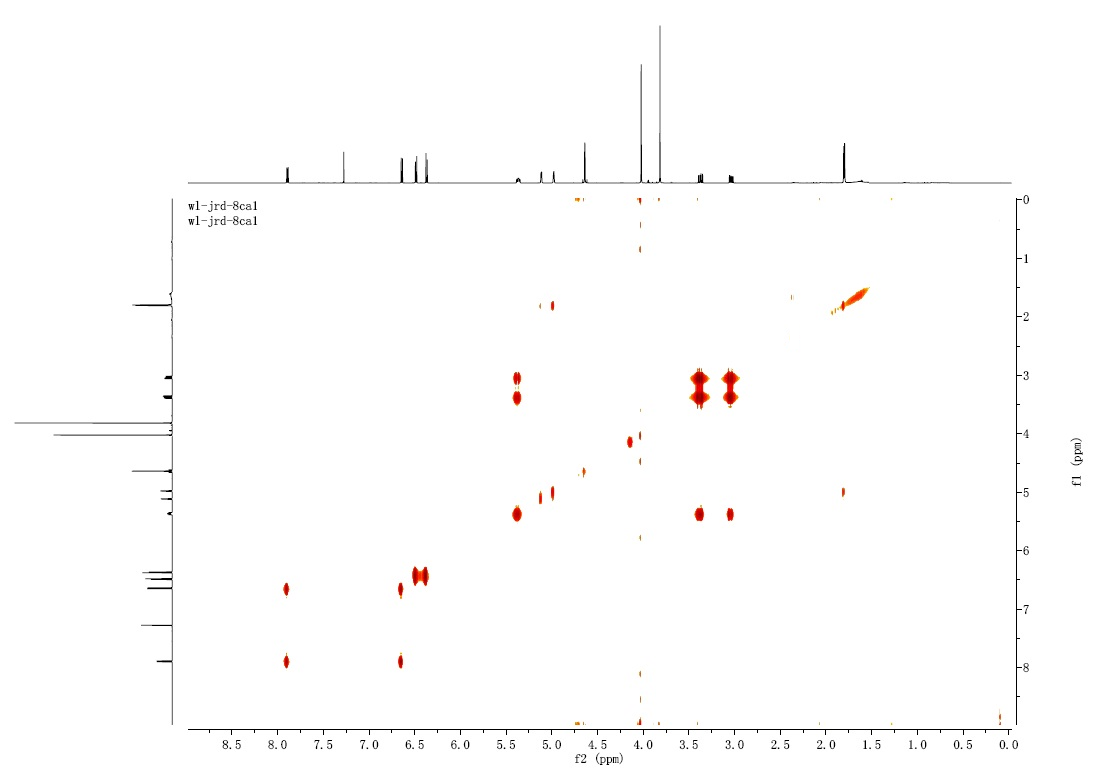


S6. ^1^H-^1^H COSY spectrum of pruinosanone A (**1**)


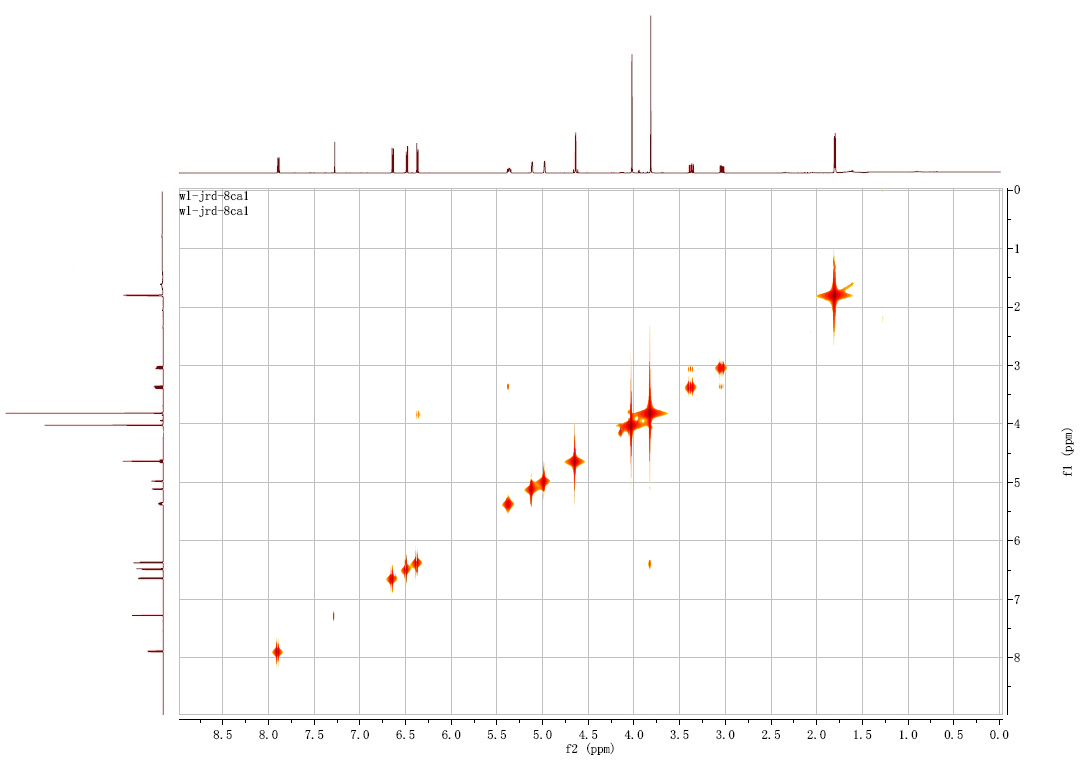


S7. NOESY spectrum of pruinosanone A (**1**)


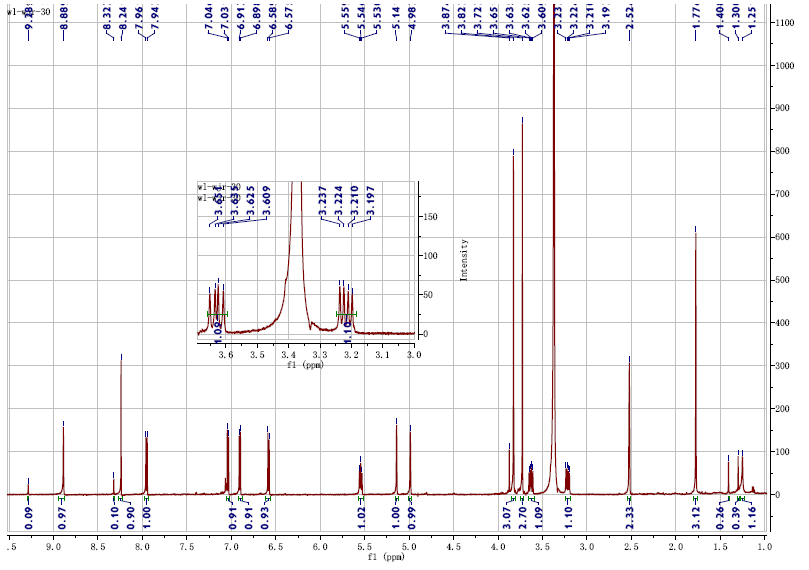


S8. ^1^H NMR (600 MHz, DMSO-*d*_6_) spectrum of pruinosanone B (**2**)

S9. ^13^C NMR spectrum (150 MHz, DMSO-*d*_6_) of pruinosanone B (**2**)

S10. DEPT spectrum (150 MHz, DMSO-*d*_6_) of pruinosanone B (**2**)

S11. HSQC spectrum of pruinosanone B (**2**)

S12. HMBC spectrum of pruinosanone B (**2**)

S13. ^1^H-^1^H COSY spectrum of pruinosanone B (**2**)


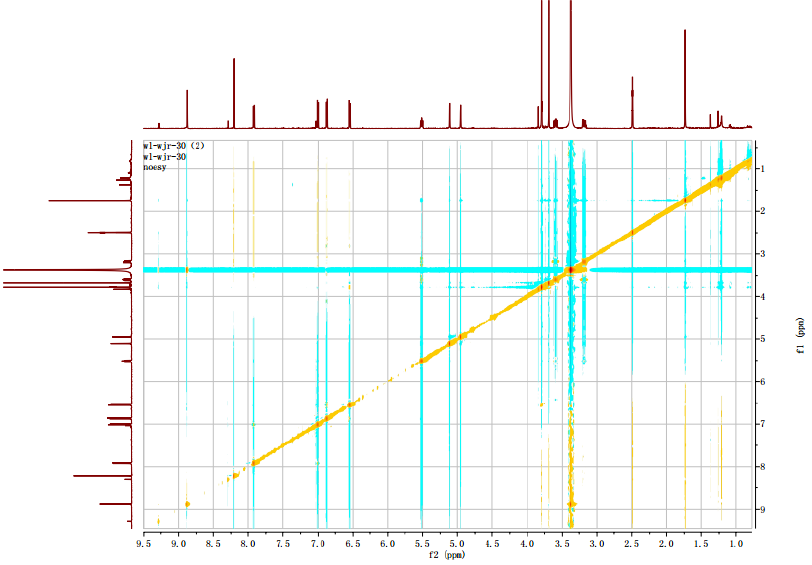


S14. NOESY spectrum of pruinosanone B (**2**)


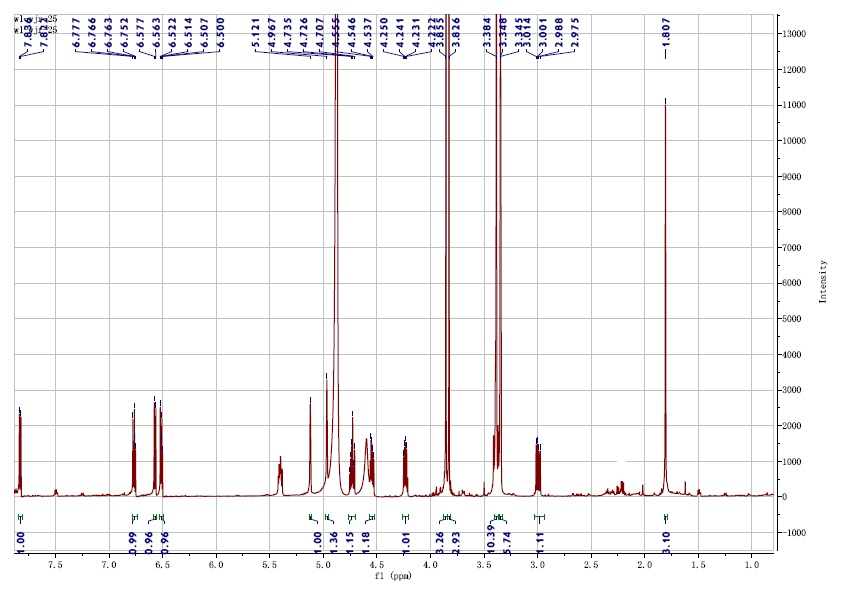


S15. ^1^H NMR (600 MHz, CD_3_OD) spectrum of pruinosanone C (**3**)

**
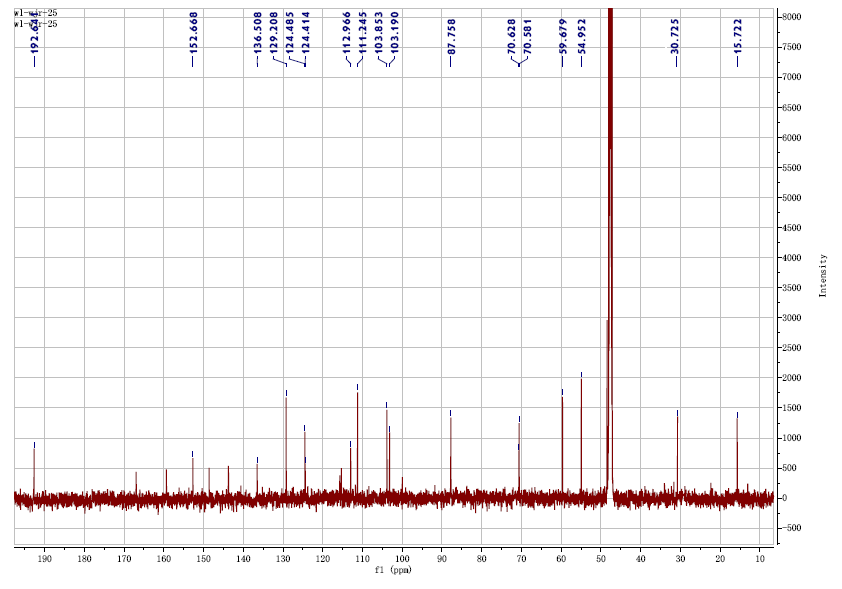
**

S16. ^13^C NMR spectrum (150 MHz, CD_3_OD) of pruinosanone C (**3**)


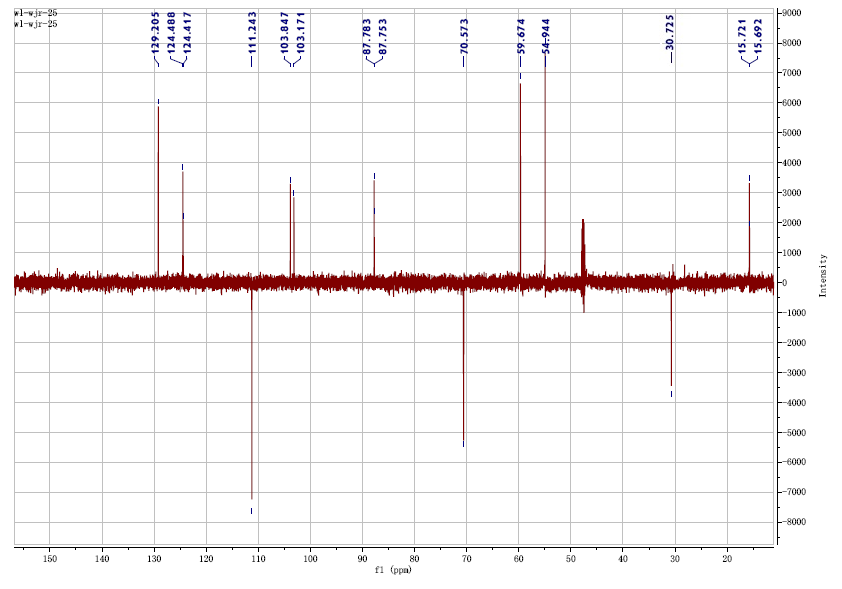


S17. DEPT spectrum (150 MHz, CD_3_OD) of pruinosanone C (**3**)

**
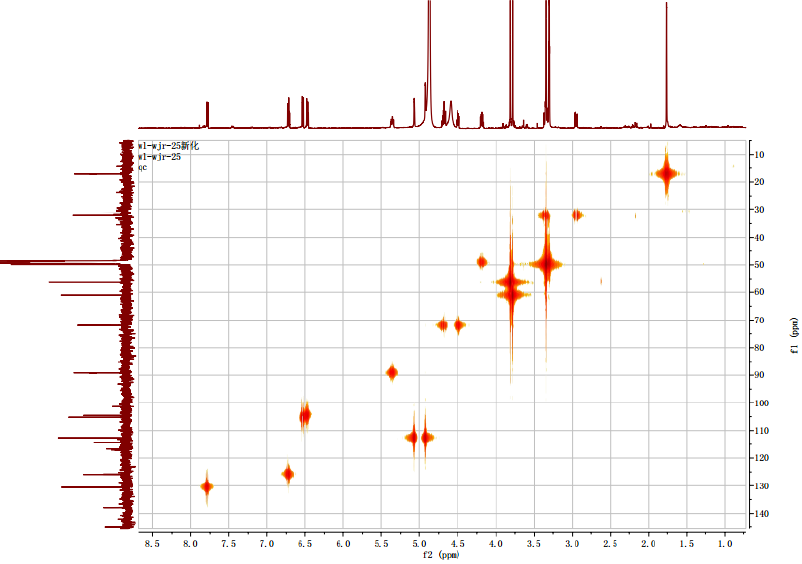
**

S18. HSQC spectrum of pruinosanone C (**3**)

**
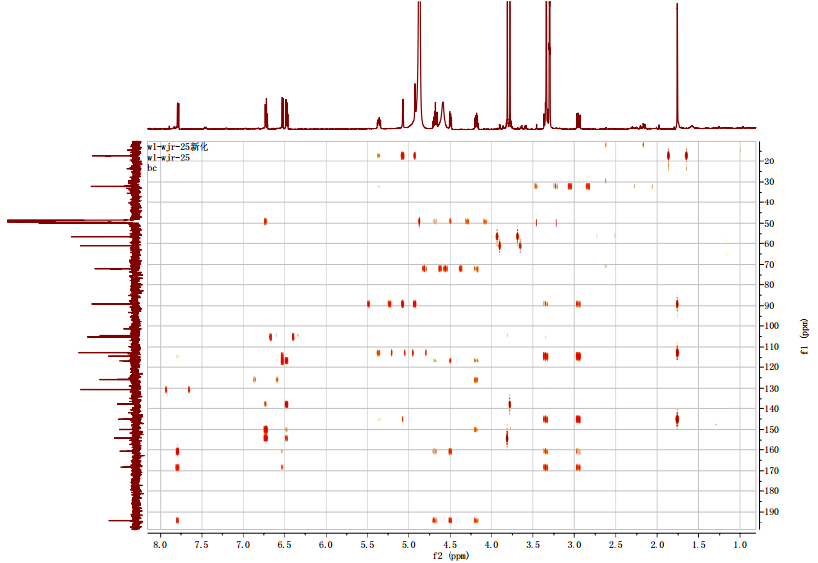
**

S19. HMBC spectrum of pruinosanone C (**3**)


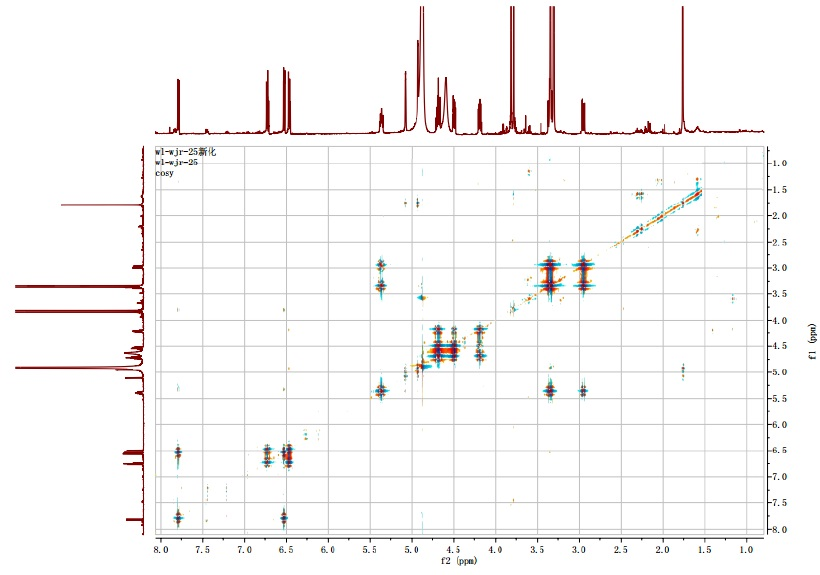


S20. ^1^H-^1^H COSY spectrum of pruinosanone C (**3**)
